# Human Liver Infection in a Dish: Easy-To-Build 3D Liver Models for Studying Microbial Infection

**DOI:** 10.1371/journal.pone.0148667

**Published:** 2016-02-10

**Authors:** Debora B. Petropolis, Daniela M. Faust, Matthieu Tolle, Lise Rivière, Tanguy Valentin, Christine Neuveut, Nora Hernandez-Cuevas, Alexandre Dufour, Jean-Christophe Olivo-Marin, Nancy Guillen

**Affiliations:** 1 Cell Biology of Parasitism Unit, Inserm U786, BCI, *Institut Pasteur*, Paris, France; 2 Hepaciviruses and Innate Immunity Unit, Department of Virology, *Institut Pasteur*, Paris, France; 3 Quantitative Image Analysis, BCI, *Institut Pasteur*, Paris, France; Universidade Federal do Rio de Janeiro, BRAZIL

## Abstract

Human liver infection is a major cause of death worldwide, but fundamental studies on infectious diseases affecting humans have been hampered by the lack of robust experimental models that accurately reproduce pathogen-host interactions in an environment relevant for the human disease. In the case of liver infection, one consequence of this absence of relevant models is a lack of understanding of how pathogens cross the sinusoidal endothelial barrier and parenchyma. To fill that gap we elaborated human 3D liver *in vitro* models, composed of human liver sinusoidal endothelial cells (LSEC) and Huh-7 hepatoma cells as hepatocyte model, layered in a structure mimicking the hepatic sinusoid, which enable studies of key features of early steps of hepatic infection. Built with established cell lines and scaffold, these models provide a reproducible and easy-to-build cell culture approach of reduced complexity compared to animal models, while preserving higher physiological relevance compared to standard 2D systems. For proof-of-principle we challenged the models with two hepatotropic pathogens: the parasitic amoeba *Entamoeba histolytica* and hepatitis B virus (HBV). We constructed four distinct setups dedicated to investigating specific aspects of hepatic invasion: 1) pathogen 3D migration towards hepatocytes, 2) hepatocyte barrier crossing, 3) LSEC and subsequent hepatocyte crossing, and 4) quantification of human hepatic virus replication (HBV). Our methods comprise automated quantification of *E*. *histolytica* migration and hepatic cells layer crossing in the 3D liver models. Moreover, replication of HBV virus occurs in our virus infection 3D liver model, indicating that routine *in vitro* assays using HBV or others viruses can be performed in this easy-to-build but more physiological hepatic environment. These results illustrate that our new 3D liver infection models are simple but effective, enabling new investigations on infectious disease mechanisms. The better understanding of these mechanisms in a human-relevant environment could aid the discovery of drugs against pathogenic liver infection.

## Introduction

The liver performs a multitude of functions in metabolism, detoxification and immune surveillance, is composed of several specific cell types, including hepatocytes and liver sinusoidal endothelial cells (LSEC) accounting for around 80% of the liver mass, and characterized by its structural and functional complexity [1]. Human liver is an important target organ for infections with pathogens of different origin [2] such as bacteria (e.g. *Listeria*), protozoan parasites (e.g. *Plasmodium* species or *Entamoeba histolytica*) and viruses (e.g. hepatitis viruses, yellow fever virus). Animal models are the main experimental systems to study hepatic infection, despite their experimental complexity, the low throughput and considerable cost, and ethical concerns. However, the relevance of animal (and especially rodent) models for human physiology is questionable, as for human inflammatory diseases [3]. Several aspects of hepatic pathogen-target cell interactions have been investigated with cell lines in standard 2D monocultures (e.g. references in review Marie and Petri, Ann. Rev. Microbiol., 2014) [4]. However, questions that can be addressed with such simple experimental systems are restricted since these cultures bear numerous limitations, notably the simplified environment (absence of physical or spatial constraints like matrix stiffness, of heterotypic cell-cell interactions), the rapid loss of the differentiated phenotype in primary 2D monocultures (e.g. LSEC and hepatocytes dedifferentiate within 72h) and the limited repertoire of hepatic functions expressed by established cell lines [5] [6].

A powerful alternative to overcome limitations inherent to *in vivo* animal models and 2D cell cultures consists in building biomimetic tissue systems (also named “organs in a dish” or micro-physiological systems). Tissue-like systems allow the use of primary or immortalized human cells, the control of the non-cellular components of the microenvironment and analysis by advanced imaging techniques. Major advantages of this approach include the reduction of the *in vivo* complexity to a controlled but still physiologically relevant level, thereby optimally adapting the experimental system, and the possibility to add or subtract specific components to define their individual roles. While in the cell biology field the utility and advantages of *in vitro* tissue-like models are recognized, for infectious disease studies they have been used only rarely [7][8].

In this work, we elaborated versatile, easy-to-build and highly reproducible human 3D liver *in vitro* cell culture models dedicated to investigate key features of hepatic infection in a context relevant for the human pathophysiology, seeking the appropriate balance between simplicity and physiological effectiveness for the goal of each study. We present four new setups based on our previously established human 3D liver model [9] that allow us to address questions that could not be investigated in previously described liver models. We describe detailed protocols for the construction of these new setups and detail their utility, validation and availability. The 3D liver models here described are reproducible and easy-to-build as they were constructed with commercially available COL I scaffold and human cell lines, taking into account the difficulties inherent to human primary cell cultures (limited availability, inter-donor phenotypic variability and stability) and the manipulation of biomaterials as cellular scaffold.

Proof-of-concept of the use of the 3D liver models for infectious disease studies was obtained from interactions with two hepatic pathogens belonging to distinct classes and causing liver diseases with high impact on public health. The extracellular protozoan parasite *E*. *histolytica* is the etiological agent of human amoebiasis, a disease leading to several thousand deaths per year. The hepatitis B virus (HBV) chronically infects 400 million people worldwide and is a leading driver of end-stage liver disease and liver cancer.

Here, we demonstrate the use of the 3D liver model setups to assess various aspects of *E*. *histolytica* liver invasion, including crossing the endothelial barrier and hepatocyte layers and 3D migration toward hepatocytes. We show that the efficiency of amoebae to invade the model is related to their degree of virulence. Compared to our previously published model [9], the new setups allow the assessment of hepatocyte layer crossing, an important process in amoebiasis pathogenesis, in which the parasite penetrates and destroys the hepatocyte plates of the parenchyma, causing amoebic liver abscess formation. Furthermore, one of the setups provides a new 3D model for human viral infection studies, since it sustains productive HBV infection.

Together the data demonstrate that the human 3D liver model setups we describe are appropriate novel tools for hepatic infection studies in a context relevant for human physiology.

## Materials and Methods

### Setup 1—Single hepatocyte layer in Collagen-I sandwich

A 3D COL-I matrix was polymerized in 35mm diameter cell culture dishes (ibidi-81156) by adding 650μl of a bovine COL-I (Nutragen, Advanced Biomatrix) solution (1.0mg/ml) in DMEM (Dulbecco’s Modified Eagle Medium 31966–047 Invitrogen) and incubating for up to 1h at 37°C in a humidified incubator at 5% CO_2_. A homogeneous suspension of cells (4x10^5^) from the human hepatoma (well differentiated) Huh-7 cell line (JCRB0403, JCRB cell bank) was carefully added on top of the polymerized matrix. Cells were incubated overnight (37°C, 5% CO_2_) in complete DMEM medium (i.e. with 10% foetal bovine serum). The medium was replaced by 150μl of the same COL-I solution. After polymerization (40min, 37°C) complete DMEM was added. The setup was used to construct setups 2–4 after overnight incubation, or for experiments after 3 days to 14 days of culture, with daily medium change.

### Setup 2—Double hepatocyte layer in Collagen-I matrix

The medium of setup 1 was replaced by 200μl of COL-I solution. After polymerization (40min, 37°C), a new layer of Huh-7 cells (8x10^5^) was plated on the top. Cultures were incubated overnight. The setup was completed with a final COL-I layer (150μl). Cultures were incubated in complete DMEM for 24h before infection. The quality of the Huh-7 cell layers (morphology of the cells, even spread, surface coverage) was examined by microscopy before each experiment. Furthermore, cell surface E-cadherin labelling was evaluated by two-photon microscopy.

### Setup 3—LSEC-double hepatocyte layer co-culture

Setup 3 was constructed as setup 2, but with a bigger volume of COL-I solution (200μl) on top. After polymerization, we added 3x10^5^ LSEC cells in complete DMEM. The LSEC used here are a human LSEC line (kindly provided by G. Grau), which was previously established by immortalization of primary LSEC cultures with human telomerase and SV40 T-antigen transgenes, and has been shown to maintain the expression of markers of corresponding primary cultures [10]. The number of LSEC cells was chosen to reach confluence after 48h of culture, as monitored by microscopic observation during the setup phase. The barrier function was tested as described [9]. The medium was changed daily and the model was used for infection when full surface coverage was reached (i.e. 48h of culture).

### Setup 4—LSEC co-culture with Huh7-NTCP hepatocytes susceptible to HBV infection

The equivalent to setup 1 was constructed with either Huh-7 or Huh7-NTCP (kindly provided by S. Urban, (Heidelberg University Hospital, Germany) cells, a Huh-7 transfectant cell line stably expressing the sodium-taurocholate co-transporter polypeptide (NTCP), the receptor for HBV [11]. LSEC (3x10^5^) were plated on the top of the polymerized COL-I layer (40min, 37°C), and the co-cultures incubated for 48h (37°C, 5% CO_2_) in complete DMEM with daily medium change. Huh7-NTCP cells were maintained in DMEM with 10% fetal calf serum.

### *E*. *histolytica* culture and invasion assay

Virulent or attenuated trophozoites from the *E*. *histolytica* strain HM1:IMSS were cultivated in TY-S-33 medium as described [12]. Virulence was maintained by regular passage of trophozoites in the hamster model [13]. The virulence attenuated HM1:IMSS strain corresponds to a long-term *in vitro* cultivated (i.e. more than 10 years in axenic culture) trophozoites, having lost the ability to induce liver abscesses in the hamster, while maintaining adhesive and cytotoxic activities when tested with standard 2D mammalian cell cultures [13].

Invasion experiments were performed as previously described [9], with green cell tracker-labelled amoebae (6x10^4^) added on top of the different 3D liver system setups. Hepatic cells were labelled with red cell tracker. After 1.5h, 3h or 6h of incubation, two-photon microscopy was used for visualization and acquisition of 3D images. The COL-I matrix fibres were detected by the second harmonic generation (SHG) signal. Images were analysed automatically.

### HBV production and infection

The HepAD38 cell line is derived from HepG2 cells and contains the HBV genome (subtype ayw) under tetracycline control [14]. HepG2 H1.3ΔX cells are derived from HepG2 cells and contain a stably integrated 1.3-fold HBV genome carrying premature stop codon mutations in both 5’ and 3’ HBx open reading frames.

Huh7-NTCP cells were infected as previously described [15]. 2D cultures or 3D LSEC-Huh7-NTCP models (setup 4) were infected with normalized amounts of virus at a MOI of 20 genome equivalents/cell in complete DMEM supplemented (except when indicated) with 4% PEG-8000. The detailed methodology used for HBV production, infection and measurement of HBV transcripts by RT-qPCR is given in the supporting information data section (S1 File).

### Automatic 3D image analysis of liver invasion

For each microscopic field (0.28mm^2^) the total number of amoebae, their z-position and the z-distance of the cell layers from the upper matrix border were determined using an automated protocol developed with the Icy software ([16], http://icy.bioimageanalysis.org) that can be found at: http://icy.bioimageanalysis.org/protocol/3D_tissue_invasion_analysis. The z-position of the centre of each amoeba was measured, and compared to the z-position of the reference layer (depending on the model, the matrix border or the LSEC layer). The invasion rate was reported using two measures: (i) the overall crossing rate, calculated as the ratio of amoebae crossing the reference layer (deeper z-position) over the total number of amoebae per field, and expressed as a percentage; (ii) the individual migration distance, measured as the relative position of each amoeba between the reference layer and the hepatocyte layer, and expressed as a percentage (0% for non-crossing amoebae, and 100% for amoebae reaching the hepatocytes). The protocol was able to analyse all experimental conditions in a fast and robust way (each condition with 10 individual fields and from 5 independent experiments) using the same parameters in a fast and robust way.

## Results

### Development of the 3D liver model setups and control of the distance between cell layers

**Fig 1:**
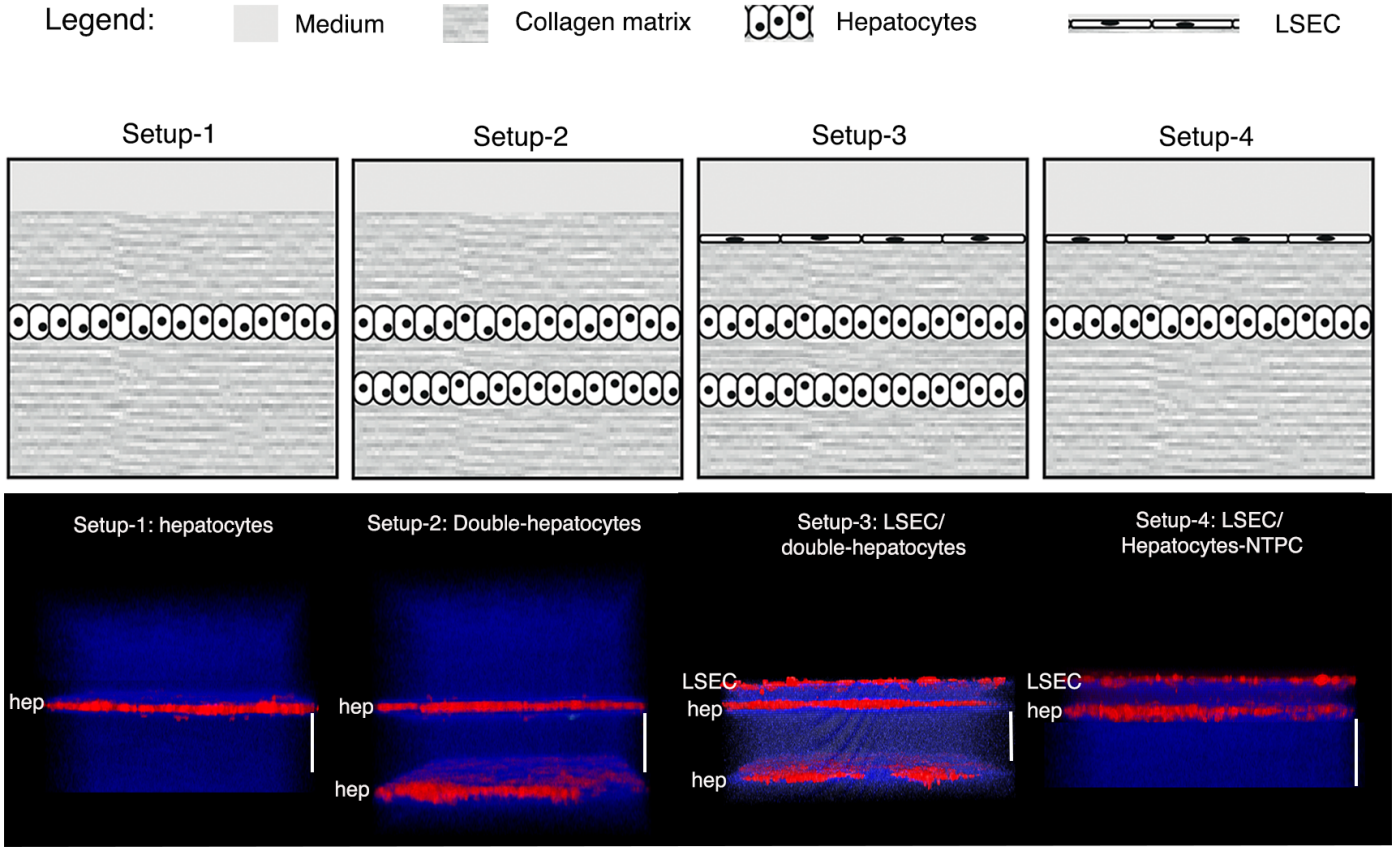
3D liver model setups developed for hepatic infection studies. The upper row of panels schematically represent the model four different 3D liver system setups, the lower row 3D reconstructions (transversal views) of the corresponding two-photon microscopy images, acquired after 3d (setup 1, 2 and 4) or 4d (setup 3) of culture. Cells were labelled with red cell tracker, COL-I fibres visualized by SHG signals (in blue). Scale bar = 100μm.

The 3D liver model design mimics the layered structure of hepatic sinusoids and comprises the main components (COL-I matrix, Huh-7 hepatocytes and LSEC). Huh-7 layers are embedded in a COL-I matrix sandwich (Fig 1) to preserve a physiological 3D environment [6]. Four optimized 3D liver model setups (Fig 1) were designed for analyses of different aspects of pathogen-hepatic cell interactions (Table 1 depicts their respective utility): pathogen migration towards hepatocytes (setup-1 and 2), crossing of the hepatocyte layer (setup-2 and 3), crossing of the LSEC endothelial barrier and the hepatocyte layer (setup 3), using the extracellular parasite *E*. *histolytica*, and to determine the rate of viral invasion and replication (setup 4), using HBV virus.

**Table 1:** Distinct 3D liver model setups for different hepatic infection study purposes.

Study purpose	Setup-1 Single hepatocyte	Setup-2 Double hepatocyte	Setup-3 LSEC-double hepatocyte	Setup-4 LSEC-hepatocyte
3D migration through ECM	√	√	√	√
Directional migration to hepatocytes	√	√	√	√
Crossing hepatocyte layers	—	√	√	—
Crossing endothelial barrier	—	—	√	√
LSEC response to the presence of pathogens	—	—	√	√

To establish the optimal setups (Fig 1 and Table 2), we tested crucial cell culture parameters as cell numbers, COL-I matrix density (1.0, 2.0 and 3.0mg/ml COL-I), time of cell adhesion (30min to overnight) and time of (co-)culture (24h to 2 weeks). Cell morphology, growth and spreading were monitored for each culture condition. Scaffolds obtained with COL I solutions of 1.0 mg/ml provided the best Huh-7 spread, producing a homogeneous distribution in a single hepatocytes layer. In contrast, in matrices of higher COL I concentrations, Huh-7 cells were less homogeneously distributed, with many areas of high cell agglomeration (data not shown). In fact, the use of 1.0 mg/ml COL I solution for the scaffold matrix turned out to be a key element for the production of 3D liver models with a well distributed single layer of hepatocytes that consequentially can mimic the liver sinusoid organization, a prerequisite for the utilization of the models for our infection studies. Note that such an organization was observed until 5 days of culture for the optimized cell numbers seeded (see Table 2). It is important to point out that the initial matrix density is strongly modified over time of culture with the hepatic cells, resulting in more dense and heterogeneous matrix [9].

**Table 2:** 3D liver model setups.

3D liver system building blocks	Setup 1 Single hepatocyte	Setup-2 Double hepatocyte	Setup-3 LSEC-double hepatocyte	Setup-4 LSEC-hepatocyte^a^
COL matrix I^b^	650 μl	650 μl	650 μl	650 μl
Hepatocytes I	4.10^5^	4.10^5^	4.10^5^	4.10^5^
Incubation	Overnight	Overnight	Overnight	Overnight
COL matrix II^b^	150 μl	175 μl	175 μl	150 μl
Hepatocytes II	X	8.10^5^	8.10^5^	X
Incubation	X	Overnight	Overnight	X
COL matrix III^b^	X	150 μl	175 μl	X
LSEC	X	X	3.10^5^	3.10^5^
Incubation	2 days	Overnight	2 days	2 days
Time to build the system	3 day	3 days	4 days	3 days

footnotes:

^a^- susceptible to HBV infection;

^b^- solution (1mg/ml)

**Fig 2:**
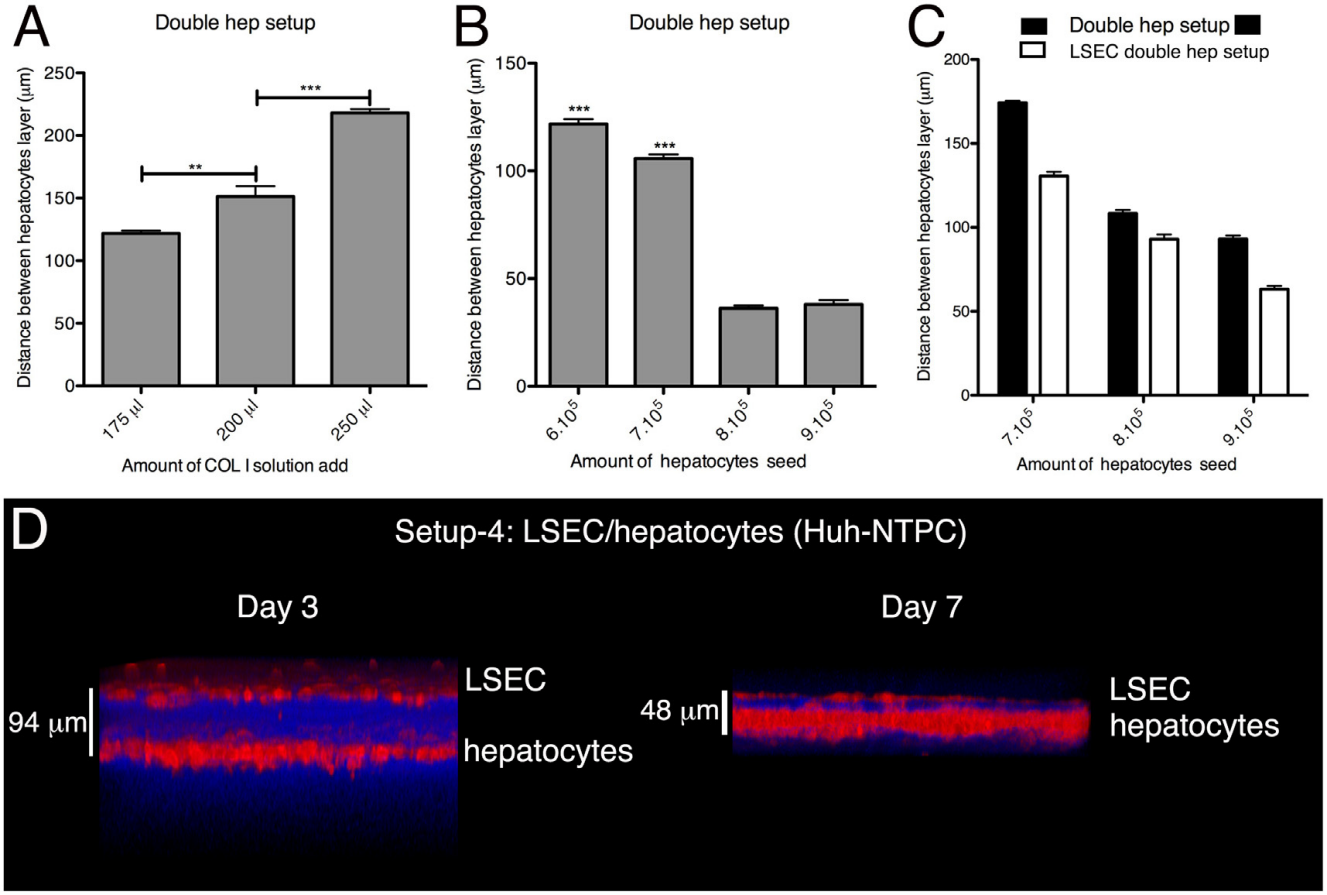
Development of the 3D liver model setups: control of the COL-I matrix height separating hepatocyte layers. (A) Double hepatocyte layer model (setup 2) built with identical cell numbers seeded and different amounts of COL-I solution used for the matrix separating the cell layers. The graph represents the distance between the two hepatocyte layers after 3d of culture. (B) The same setup 2 with different cell numbers seeded at the up hepatocytes layer but identical volumes of COL-I solution (175ul). Graphic representation of the distance between the two hepatocyte layers after 3d of culture. (C) Setup 2 seeded with indicated numbers of hepatocytes, co-cultured or not with an LSEC layer (setup 3). The graph shows the distance between the two hepatocyte layers. A-C) data obtained from 3 independent experiments. Standard deviation and statistical significance indicated, with ** = p < 0.01 and *** = p < 0.001. (D) 3D transversal reconstruction (ICY software) of two-photon microscopy acquisitions of LSEC/single hepatocyte layer 3D liver model (setup 4) after 3d or 7d of culture. Cells were labelled with red cell tracker, COL-I fibres visualized by SHG signals (in blue).

The distance between the cell layers was defined by the amount of COL-I solution used for the intermediate matrix layers (Fig 2A and Table 2) and the number of cells seeded (Fig 2B and 2C). The height of the matrix layer also depended on the cell types present in the setup and was lower in the presence of LSEC (Fig 2C), demonstrating differences in the matrix remodelling. Over time of culture the space separating the cell layers significantly diminished, in particular between LSEC and Huh-7 hepatocytes for which it reached less than 50 μm after 5d (Fig 2D).

To quantify the LSEC or Huh-7 layer crossing (setup 2 and 3) by *E*. *histolytica* (mean diameter of amoeba 20–50μm) and the amoebic migration in the 3D matrix towards the Huh-7 cells (setup 2), we used a mean distance of around 100μm between cell layers, enabling the distinction of the compartments. To determine the rate of viral invasion and replication, using HBV (setup 4), we used the less than 50μm distance, which better reproduces the dimensions of the Disse's space (less than 10μm) separating the two cell types *in vivo* (Fig 2D).

### Single hepatocyte layer model to analyse migration towards hepatocytes (setup 1)

The most simplified 3D liver model constructed consisted in a single Huh-7 cell monolayer (human hepatocytes model) embedded in a COL-I matrix (setup 1 in Table 1 and Fig 1). We have previously shown that culturing Huh-7 cells in a 3D COL-I matrix improved their hepatic phenotype compared to standard 2D conditions [9].

Utility: This setup is conceived for the quantitative analysis of the 3D migration of pathogens towards hepatocytes that may be a characteristic of many hepatotropic pathogens and may also be relevant for the migration behaviour of certain cancer cell types.

**Fig 3:**
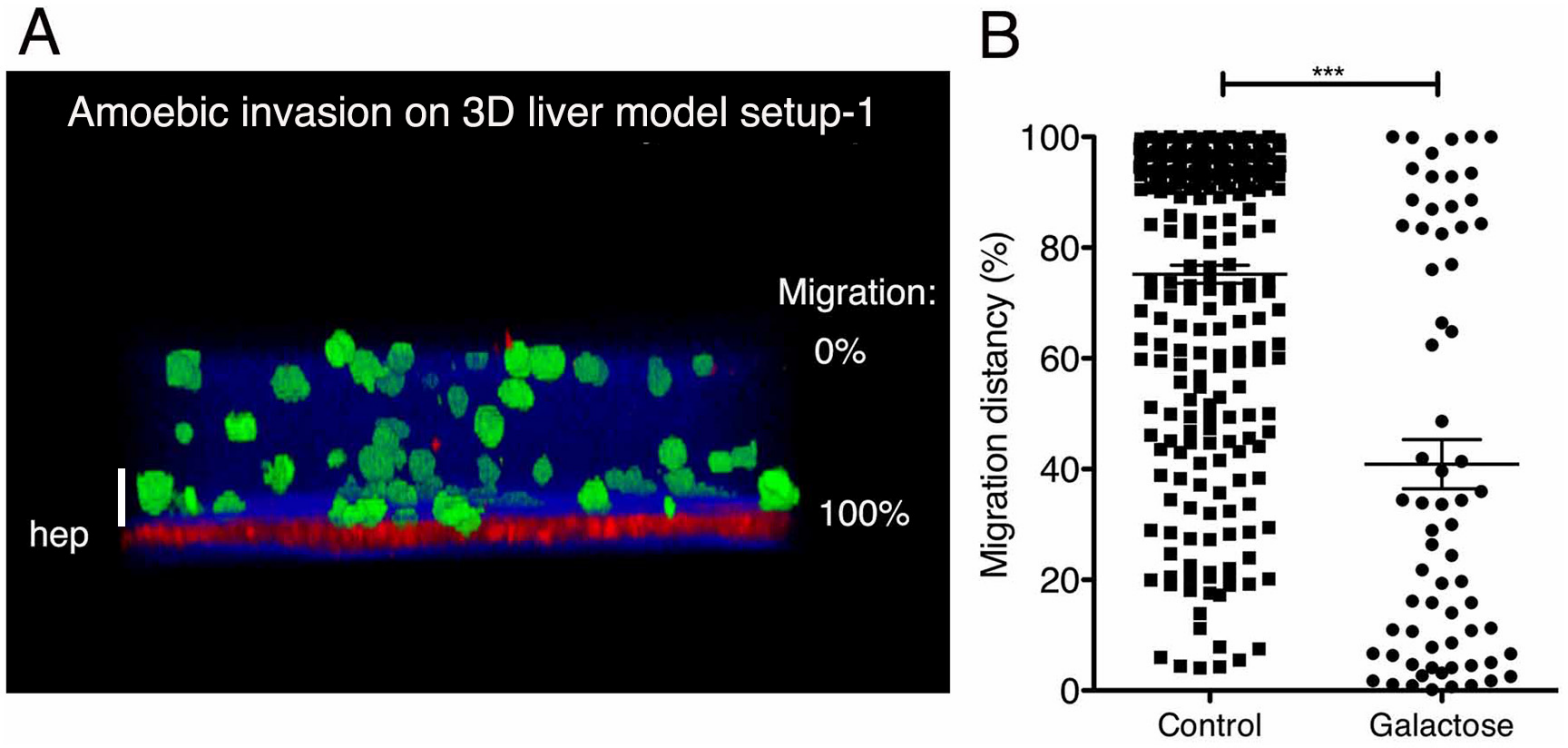
3D liver model with single hepatocyte layer: visualization and quantification of the 3D directional migration of *E*. *histolytica*. (A) 3D transversal reconstruction (ICY software) of a two-photon microscopy image of 3D liver system setup 1 at 3h after addition of virulent trophozoites on top of the model. Hepatocytes labelled with red cell tracker, amoebae with green cell tracker. COL-I fibres visualized by SHG signal (in blue). (B) Quantification of the directional migration of amoebae towards the hepatocyte layer, in the presence of 100mM glucose (control) or galactose. The migration distance (i.e. migration towards hepatocytes) was obtained by considering the amoeba position relative to the position of the matrix top and the hepatocyte layer, set as 0% and 100%, respectively (indicated in A). For each condition, 10 fields of 5 independent experiments were examined. Mean values with standard deviation and statistical significance indicated, with *** = p < 0.001. Scale bar = 50μm.

Validation: To investigate infection with *E*. *histolytica*, the setup was incubated for 3h with virulent trophozoites (Fig 3) and 3D images of different fields were taken using two-photon microscopy (Fig 3A). Images were used to quantify the amoebic migration towards the Huh-7 cells. The z-position of each amoeba was determined (ICY software) and normalized considering the position of the hepatocytes as 100% and the matrix top border as 0%. The analysis shows that the majority of the trophozoites migrated (mean around 80%) and reached the human cell layer (Fig 3B). The presence of galactose (a well-known inhibitor of *E*. *histolytica* adhesion) significantly reduced the distance migrated (mean around 40%) by the amoebae (Fig 3B), revealing an interesting correlation between cell adhesion properties and migration during *E*. *histolytica* hepatic invasion. The results provide the proof-of-concept for the use of the single hepatocyte layer setup to study 3D migration of hepatotropic parasites through ECM towards hepatocytes.

We also monitored the behaviour of amoebae inside COL-I matrices without hepatic cells and observed that amoebic viability is extremely reduced under these conditions, pointing out that the presence of hepatic cells is crucial for maintaining *E*. *histolytica* viability in the 3D liver model (i.e. in mammalian cell culture medium without serum). This result is in line with previous data demonstrating that the presence of the hepatic cells in the model changes medium composition and scaffold structure [9].

### Double hepatocyte layer 3D liver model for quantitative analysis of hepatocyte layer crossing (setup 2)

**Fig 4:**
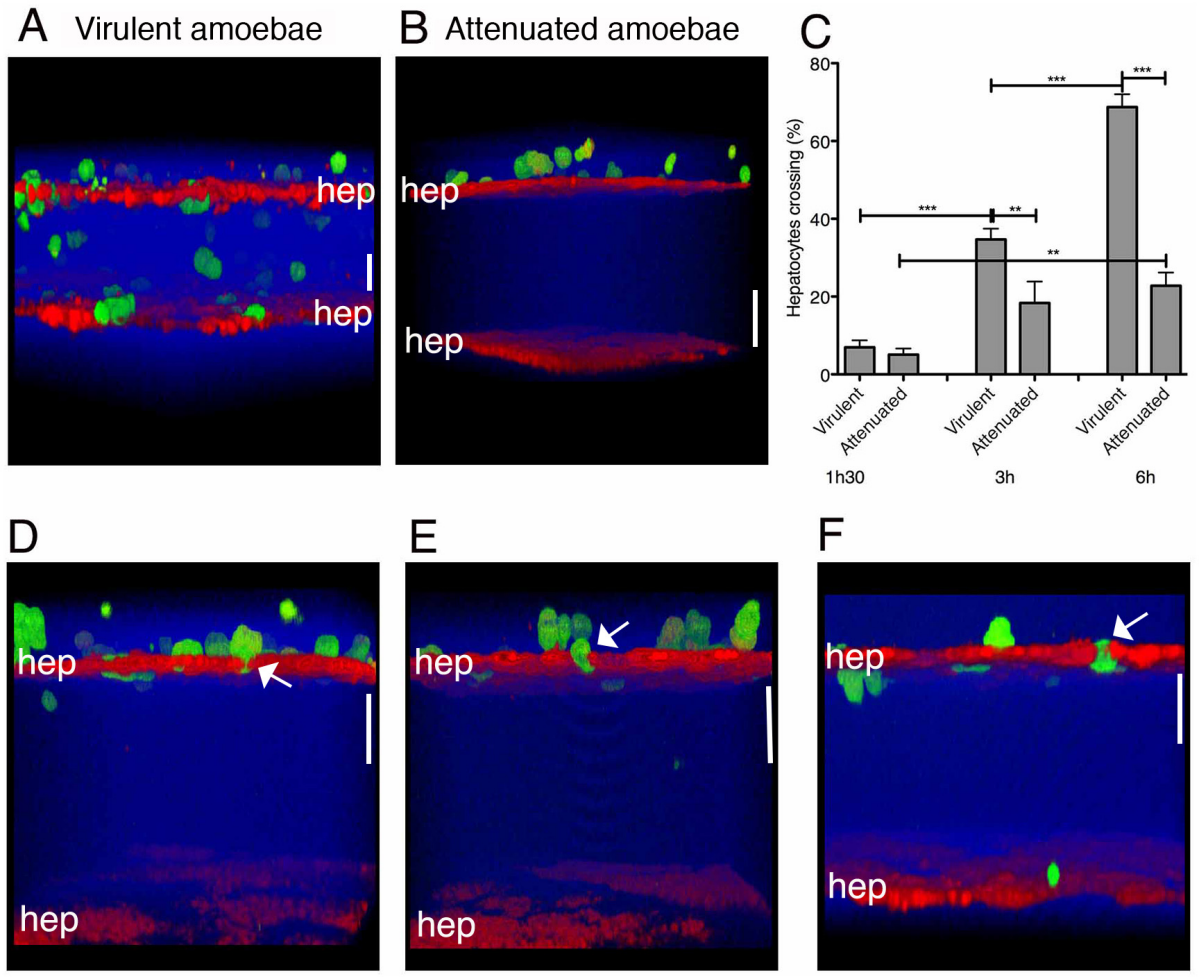
3D liver model with the double hepatocyte layer: visualization and quantification of hepatocyte crossing by *E*. *histolytica*. For two-photon microscopy acquisitions, hepatocytes were labelled with red and amoebae with green cell tracker. Col-I fibres were visualized by SHG signal (in blue). (A-B) 3D transversal reconstruction (ICY software) of 3D liver model setup 2 after 3h of interaction with virulent (A) or virulence-attenuated (B) trophozoites. (C) Quantification of the hepatocyte crossing by *E*. *histolytica* after 1.5h, 3h and 6h of interaction with the model. Obtained from 3 independent experiments. Standard deviation and statistical significance indicated, with ** = p < 0.01 and *** = p < 0.001. D-F) 3D transversal reconstruction (ICY software) enabling visualization of amoebae squeezing in between hepatocytes during hepatic crossing at 1.5h, 3h and 6h of interaction with the model (arrows). Scale bar = 50μm.

Utility: An important aspect of liver infection is the spreading of pathogens and immune cells through the parenchyma. Setup 2 of the human 3D liver model is the first one suitable to record the kinetics and to quantify the crossing of hepatocyte layers by *E*. *histolytica* (Table 1 and Figs 1 and 4). The setup consists of two Huh-7 layers in a sandwich organization with a COL-I matrix scaffold (setup 2, double hepatocyte 3D liver model, Table 1). To evaluate the presence of cell-cell adhesion between Huh-7 cells in the hepatocytes layers, we examined E-cadherin labelling by two-photon microscopy. Huh-7 cultured in this 3D liver model setup presented positive cell surface labelling for E-cadherin in all fields examined (data not shown). This observation, together with the morphological observations of the cells, are corroborated by our previous results showing that Huh-7 layers in the 3D liver model were not permeable for 1μm diameter beads [9] and thereby demonstrating that these layers form a cellular barrier at our 3D liver models culture conditions.

Validation: Whereas after 1h30 of interaction less than 10% of the virulent trophozoites crossed the top Huh-7 hepatocytes layer, the percentage significantly increased over time and reached more than 60% after 6h (Fig 4A and 4C). In contrast, almost no trophozoites crossed the bottom Huh-7 hepatocytes layer (close to 0%) at the same time points (data not shown). Virulence-attenuated amoebae (unable to produce liver abscesses in the hamster) presented a significantly reduced crossing activity at 3h (around 40% reduction) and 6h (around 70% reduction) of interaction with the 3D liver model (Fig 4C), demonstrating that the double hepatocyte setup can be used for a comparative quantitative analysis between different amoeba strains.

Moreover, morphological changes occurring in the Huh-7 hepatocyte layer during amoebic crossing can be monitored with the double hepatocyte layer setup (Fig 4D–4F). We notably observed that *E*. *histolytica* squeezed in between Huh-7 cells and almost none of them detached (arrows Fig 4D–4F).

### LSEC—double hepatocyte layer model for quantitative analysis of LSEC and hepatocyte layer crossing (setup 3)

Utility: The LSEC co-culture with two Huh-7 hepatocyte layers was the most complex 3D liver model developed (Table 1, Fig 1) and allowed the quantification of the crossing by *E*. *histolytica* of both, the LSEC and the Huh-7 layer, representing the endothelial and the hepatocyte barriers of the hepatic sinusoid. We previously demonstrated that efficient LSEC crossing by amoebae requires the presence of an underlying Huh-7 layer [9], suggesting that *E*. *histolytica* needs attraction by a hepatocytic layer to stimulate its cell layer crossing behaviour. Although this hypothesis could not be evaluated with the previously published model, it could now be addressed with the newly developed 3D liver model setups. In this work we demonstrated that the amoebas were able to cross the top Huh-7 hepatocyte layers in the 3D liver model setup 2 and setup 3 but were not able to cross the bottom hepatocyte layers.

**Fig 5:**
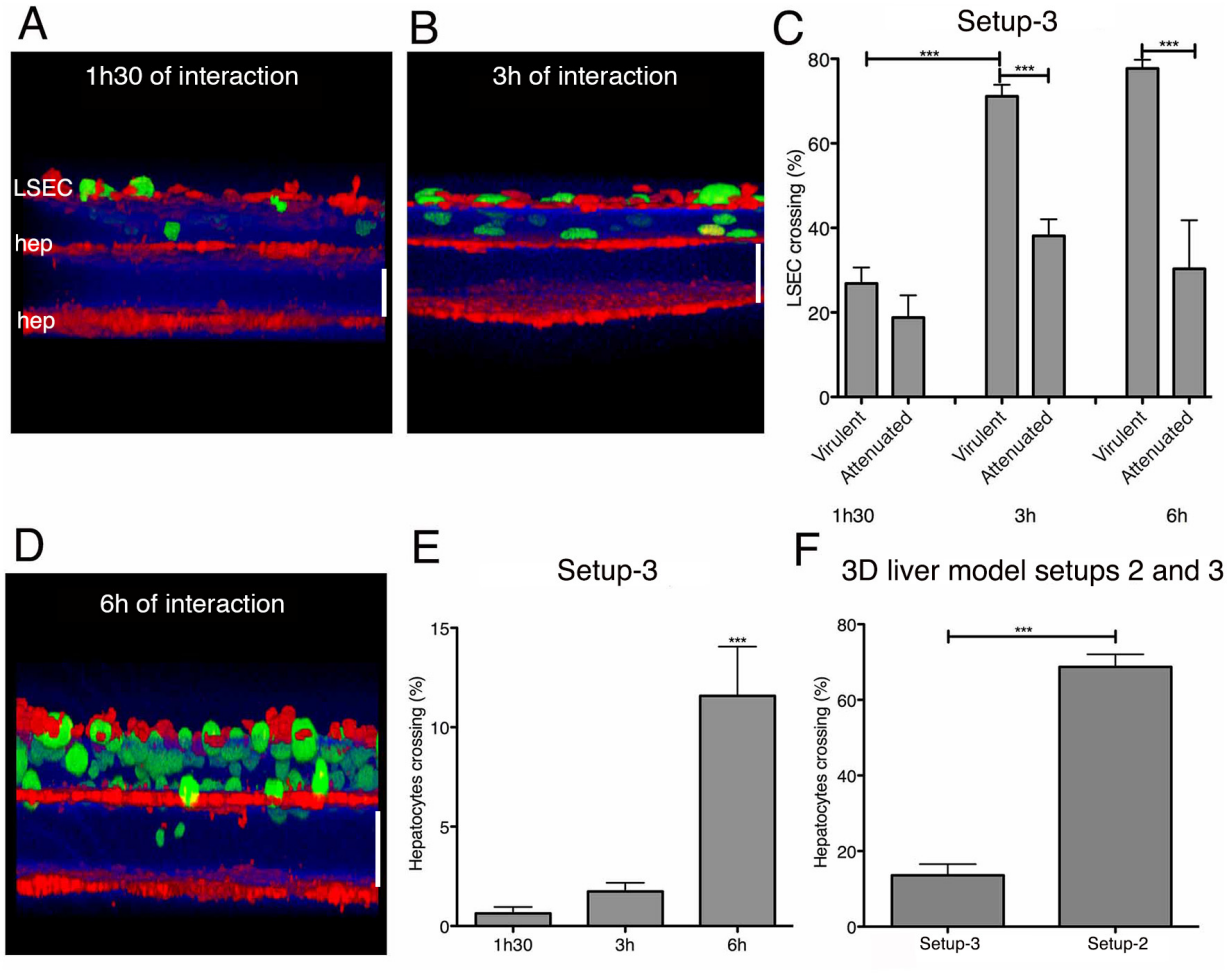
3D liver model with LSEC and double hepatocyte layers: visualization and quantification of LSEC and hepatocytes crossing by *E*. *histolytica*. For two-photon microscopy acquisitions, hepatocytes were labelled with red and amoebae with green cell tracker. Col-I fibres were visualized by SHG signal (in blue). 3D transversal reconstruction (ICY software) of images acquired at 1.5h (A) and 3h (B) of virulent amoebae interaction with the model. (C) Quantification of LSEC crossing by virulent and virulence-attenuated *E*. *histolytica*. (D) 3D transversal reconstruction (ICY software) of image acquired after 6h of interaction, visualizing amoeba crossing of the upper hepatocyte layer. (E) Quantification of the hepatocyte layer crossing by *E*. *histolytica* at indicated time-points of interaction. (F) Amoebic hepatocyte crossing rate in the LSEC/double hepatocyte layer (setup 3) and double hepatocyte layer system (setup 2, i.e. without LSEC). (C), (E) and (F) obtained from 3 independent experiments. Standard deviation and statistical significance indicated, with *** = p < 0.001. Scale bar = 100μm.

Validation: With virulent trophozoites, the LSEC crossing rate was around 20% after 1h30 of interaction and reached almost 80% after 3h and 6h (Fig 5A–5C), whereas the upper Huh-7 layer was only significantly crossed after 3h and 6h, with a crossing rate of around 10% (Fig 5D–5E). Virulence-attenuated amoebae showed a significantly lower LSEC crossing rate (around 50% lower) at 3h and 6h (but not at 1h30) (Fig 5C) and were not able to cross the Huh-7 layer.

The percentage of amoebae crossing the hepatocyte barrier was around six times lower in the presence (setup 3) than in the absence of LSEC (setup 2) (Fig 5F). This result corroborates the LSEC barrier function described previously during *E*. *histolytica* invasion of the 3D liver model [9].

Together the results using the 3D liver model setup 3 demonstrate for the first time the ability of *E*. *histolytica* to cross both hepatic sinusoid barriers (LSEC and hepatocyte layers) in an human *in vitro* model and indicate the need of an underlying hepatocyte layer to activate the *E*. *histolytica* crossing (invasive behaviour) of LSEC and hepatocyte layers.

### 3D liver model susceptible to HBV infection (setup 4)

Utility: To study HBV infection in our 3D liver model (single hepatocyte and LSEC monolayer; setup 4 in Table 2 and Fig 1), we replaced Huh-7 (not susceptible to the virus) by Huh7-NTCP cells stably expressing NTCP, which has been identified as a receptor for both HBV and hepatitis D virus [17]. The expression of NTCP in human hepatocytic cell lines (HepG2 and Huh-7) renders them susceptible to HBV infection [11].

**Fig 6:**
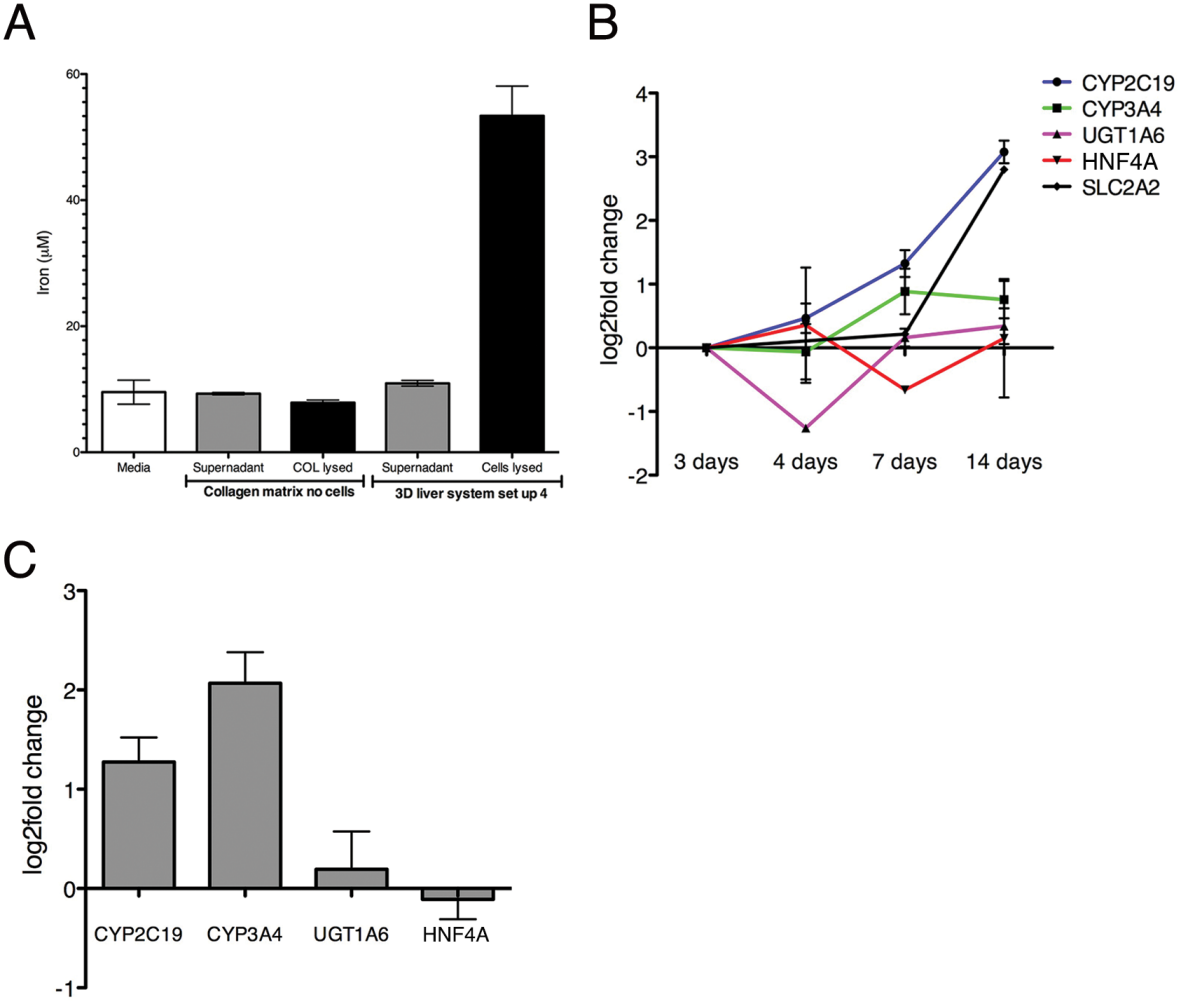
Hepatic marker expression in the 3D liver model (setup 4) with a single Huh7-NTCP hepatocyte and LSEC layer. (A) Iron accumulation in hepatocytes cultivated in the 3D liver system setup 4 for 3 days. Iron concentration in cell culture supernatant and in hepatic cells lysates. Medium alone and cell-free COL-I matrix (soluble and lysed pellet fraction) used as controls. (B) Expression level of transcripts for selected hepatic markers between 3-14d of culture, determined by q-PCR, and represented as fold change compared to the respective levels observed at 3d. (C) Hepatic marker expression in the 3D liver model with Huh7-NTCP compared with the 3D liver system with not transfected Huh-7 cells. Transcript levels determined by q-PCR and presented as fold change detected for Huh7-NTCP. Data obtained from three independent experiments. Standard deviation indicated.

Validation: We first evaluated several physiological functions of Huh7-NTCP cells in the liver 3D model. Cells cultivated for 3 days in the setup presented a strong accumulation of iron (Fig 6A) and sustained or even increased expression of hepatic markers (for cytochrome C CYP3A4, CYP2C19, transporters SLC2A, and UGT1A6, and HNF4A, key transcription factor transcript levels determined by RT-qPCR) over a 14 days period of culture (Fig 6B). Interestingly, the expression of cytochromes CYP2C19 and CYP3A4 (important functions for drug metabolism) was significantly higher in the models with Huh7-NTPC compared to untransfected Huh-7 cells (Fig 6C). We previously demonstrated that expression of most of these markers (CYP2C19, SLC2A, SLC2A1, UGT1A6) and human albumin release were significantly higher in the 3D liver model when compared to standard 2D cultures [9]. Together the results demonstrate the physiological relevance of the 3D liver model.

**Fig 7:**
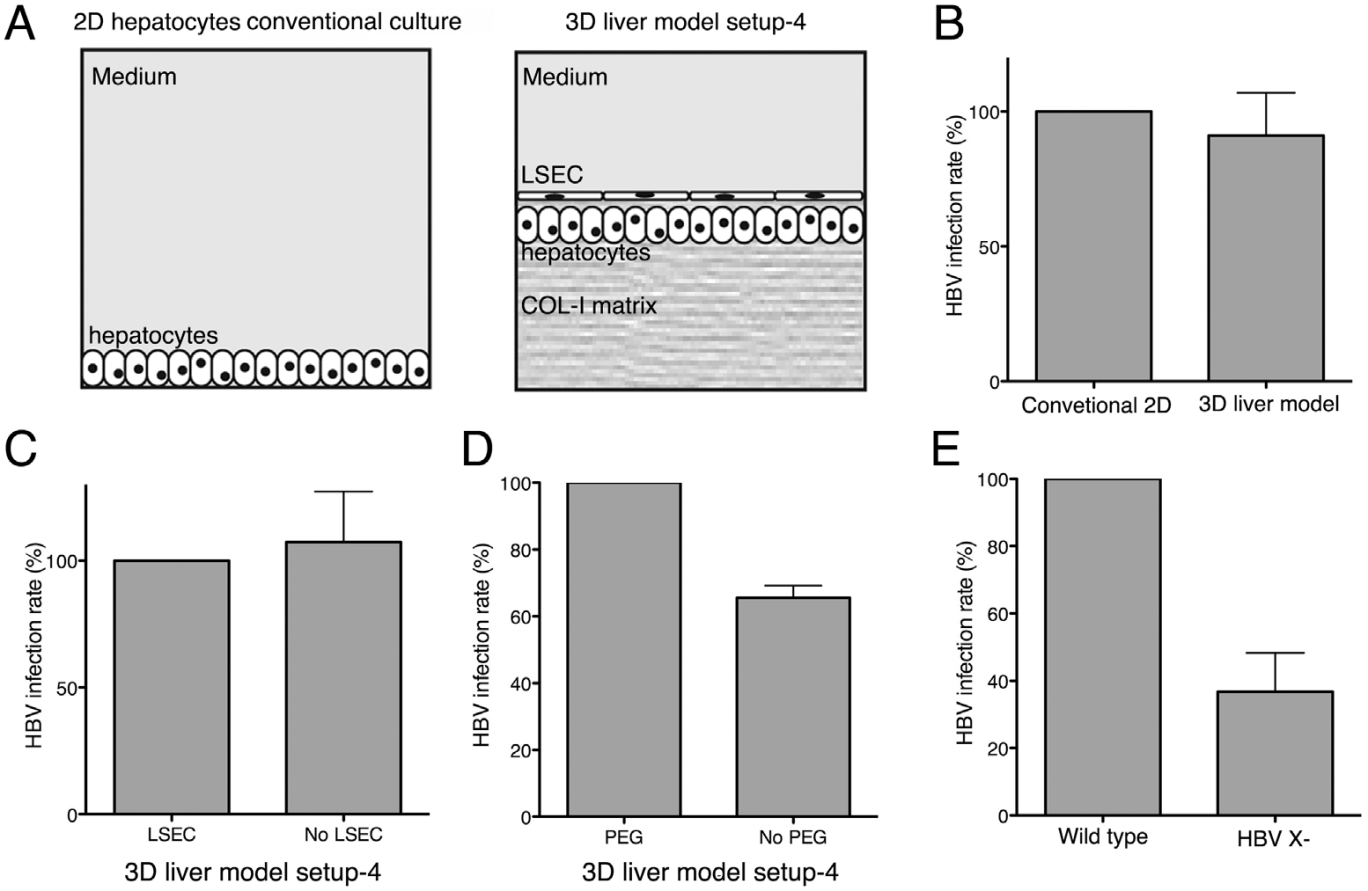
HBV infection rate at the 3D liver model setup 4. (A) Schematic representation of 2D conventional hepatocyte cultures and the 3D liver system used for HBV infection experiments. (B-E) Cells were infected with either HBVwt or HBV X- as indicated at MOI of 20 genome equivalents/cell. Total RNA was extracted 4 days after infection and HBV infection level was analysed by measuring HBV transcription by RT-qPCR. (B) Infection rates obtained with conventional 2D cultures and on the 3D liver system. (C) HBV infection rate for the 3D liver model with Huh7-NTCP upon co-culture with LSEC (setup 4) and in the absence of LSEC (setup 1) (D) or in the presence or absence of PEG. (E) 3D liver model HBV infection rate compared between wild type and HBV X- particles. Data obtained from two independent experiments. Standard deviation indicated.

For HBV infection, we chose a distance between the two cell layers of the model lower than 50μm (since HBV particles are small (42nm diameter)) (Fig 2D), which is closer to the physiological distance in hepatic sinusoids. The virus particles were added on the top of the 3D liver models, or standard 2D Huh7-NTPC cultures as controls (Fig 7A). Replication rates were evaluated after 4 days of interaction by measuring HBV transcription, using RT-qPCR, as indicator of productive infection. Similar HBV replication rates were observed with the 3D liver model and 2D cultures (Fig 7B), indicating that 3D liver model components did not function as barriers for HBV. HBV infection rates in the 3D model were similar in the presence or absence of LSEC (Fig 7C), as expected from the virus particle size and the LSEC phenotype (fenestrated cells without tight junctions) [2]. However, the presence of LSEC is essential for the matrix architecture, hepatocyte differentiation and the pro-inflammatory responses against pathogens [9].

We then evaluated the need of PEG-8000, commonly used in HBV infection protocols [18]. For Huh7-NTCP 2D cultures, PEG significantly increased the HBV infection rate (Fig 7D). In contrast, PEG addition to the 3D model had no significant effect on the viral infection efficiency. To further validate the 3D liver model for HBV infection, we studied the replication of a virus deficient for the expression of the regulatory protein HBx (X-virus), a protein essential for virus replication in *vivo* [19] [20] [21]. Infections of the 3D liver model with HBV X- virus resulted in a 60% reduction of the replication rate compared with the wild type HBV (Fig 7E), as observed for 2D culture and *in vivo* infections.

Together the results demonstrate that the Huh7-NTCP 3D liver model is an *in vitro* micro-physiological environment susceptible to HBV infection and provide the proof-of-concept for the potential use of this model to study HBV virulence factors and effects of drugs.

## Discussion

The use of human *in vitro* tissue-like models for infectious disease studies can promote the discovery of human-specific molecular mechanisms distinct from those revealed with animal models. Human tissue-like models can thus contribute to fill the gap between the knowledge acquired from animal models and 2D cultures of human cells. In the context of infectious liver diseases, a major requirement for such tissue-like models is to mimic the hepatic sinusoid architecture, thus enabling the study of the invasion events in a realistic sequence. Most of the existing 3D liver models take the hepatic cell physiology into account, but not the tissue architecture (e.g. 3D spheroid cultures), and thus can be appropriate for toxicology or drug screening purposes [22], but not to study infectious diseases. For the wide use of 3D liver models in infectious disease research, a further consideration is the accessibility of the starting material and the ease of handling. For instance, micro-tissues constructed on microfluidics devices (organ on-a-chip) can increase the system physiology in many conditions [23] [24] but also the complexity of construction (requiring lithography or microfluidics know-how), thus limiting their use.

A major consideration is the choice of the source of the human cell types, i.e. established cell lines versus primary cell cultures or iPS-derived hepatocytic cells. Cell lines generally do not fully reproduce the phenotype of differentiated cells, but are easy to cultivate in a reproducible manner. Patient-derived hepatocyte primary cultures are difficult to obtain (legal permissions, availability) and show large phenotypic variability (between different donors and between fresh and frozen cells), making their use for the infectious disease field challenging. Moreover, as previously demonstrated [5][6], *in vitro* cultured primary hepatocytes do not necessarily fully reproduce a differentiated phenotype, such that maintaining adult hepatic markers of primary hepatocytes in 2D *in vitro* cultures is extremely challenging. Human primary hepatocytes cultured as 3D spheroids or inside microfluidics chambers have given good physiological results, but both approaches are not suitable for the infectious disease field. The alternative of producing human induced pluripotent stem cell (iPS)-derived hepatocytic cells [25][26] also requires time and cell growth culture conditions that are technically challenging.

In this work, we established easy-to-build human 3D liver *in vitro* model setups customized to investigate several early aspects of hepatic infection relevant for the human pathophysiology. Using these setups, we visualized and quantified the efficiency of cell barrier crossing and intra-tissular migration of *E*. *histolytica*. The double hepatocyte layer setups (setup 2 and 3) represent the first experimental tools allowing quantification of the hepatocyte layer crossing activity of this parasite. We demonstrated that for *E*. *histolytica* crossing of a hepatocytic cell layer, it is necessary another underlying hepatocytic layer, suggesting parasite attraction by the latter that activates the invasive behaviour of the amoebae. We also showed that migration and barrier-crossing efficiency are related to amoebic virulence and observed amoebae passaging between individual hepatocytes of the layer. These results are in line with the previously described E-cadherin signal reduction during amoebic interaction with hepatocytes, suggesting changes in tight junction organization that may imply weakening/disruption of cell-to-cell contacts. We concluded that the mechanism of hepatic epithelium invasion differs from the mechanism allowing liver endothelium crossing, in which the presence of *E*. *histolytica* mainly induces LSEC retraction and cell detachment [9]. Taken together, our results validate the new 3D liver model setups as novel tools able to open new avenues for *in vitro* studies of liver invasion by pathogens. The better understanding of the molecular mechanisms sustaining hepatic spread could lead to the discovery of drugs protecting the liver against pathogen infection.

One of our objectives was the construction of a simple and fast 3D liver model for HBV infection. Human cells of the HepaRG line and of hepatocyte primary cultures are the most common options for *in vitro* HBV infection studies. However, use of these cell types for the 3D liver model presents several difficulties. Only differentiated HepaRG cells sustain HBV infection [27], and cell differentiation is achieved with a time-consuming culture protocol (around 4 weeks). Considering the advantages of availability, reproducibility, phenotypic stability, and speed, we chose to work with stably transfected Huh-7 cells expressing NTCP. We show that our model sustains productive HBV infection of hepatocytes, at a level comparable to the infection rate in 2D cultures. Like in 2D cultures and in *in vivo* experiments, HBx factor is also required for productive HBV infection in the 3D liver model. The data further indicate that the LSEC layer did not function as a barrier for the virus, as expected from the size of the virus particle (around 42 nm) and the LSEC phenotype (presence of fenestrations of around 100nm diameter and absence of tight junctions [2]). One crucial aspect for disease progression is the pathogen-induced (pro- or anti-) inflammatory tissue response, such as the IFNα and IFNγ production upon HBV infection. The experimental system described here can be used for future cytokine profiling during viral infection, as we have already characterized the cytokine release upon *E*. *histolytica* infection in the original 3D liver model [9].

It is important to consider the physiological limitations inherent to our 3D liver model, before adapting it for other scientific purposes. The Huh-7 cell line, like other hepatocyte *in vitro* models, expresses only a limited panel of adult hepatic functions. However, we have previously shown that several hepatic markers remain expressed on our 3D liver model [9]. Moreover, we here demonstrate that Huh7-NTCP cells cultured in the 3D liver model configuration accumulate iron and express markers related to liver physiology. These results together strongly suggest that our setups are physiologically more relevant than standard 2D cultures for many questions related to infectious diseases, and this represents the main advantage of using the 3D liver infection model. Furthermore, the LSEC-hepatocyte cross-talk that is known to be essential for many liver sinusoid physiological responses is ignored by experiments using traditional 2D models.

Our setups were primarily designed for the characterization of parasite—human cell interactions and a main advantage of our approach is the mimicking of the layered hepatic sinusoid structure allowing the direct contact between parasite and LSEC. Moreover our approach is easy to handle, scalable and highly reproducible, since built with established human cell lines and commercially available COL I.

Our models provide a platform that can be easily extended, for example by the inclusion of other hepatic cell types such as immune-competent Kupffer cells or stellate cells. However it should be noted that human cells of these two cell types are difficult to obtain and short-lived in primary cultures, and to our knowledge, human Kupffer cell lines have not yet been established. Their addition may open new research applications of the model, but will require detailed investigations of their effects on the model and the definition of the optimal setup.

Another technical aspect that could be adapted for further applications is the matrix thicknesses. To analyse *E*. *histolytica* migration and interaction with LSEC or Huh-7 cells, we used matrix thicknesses that clearly separate the hepatic cell layers. Further adaptations of the model (for questions different from the migration of cells as large as amoebae) may include reduction of the matrix thickness to allow direct heterotypic interactions between the two cell types. However, aiming for thinner ECM layers (below 10 μm) would considerably increase the complexity of the model construction, and biochemistry and/or bioengineering knowhow/techniques would become essential.

In conclusion, the newly developed setups of our human 3D liver model are easy-to-build alternative tools for hepatic infection studies. Our *in vitro* models offer a fast way to assess many poorly studied questions in an environment that is more physiological and relevant for the human infectious diseases.

## Supporting Information

S1 File Supporting Information. Method details on the HBV production and infection, RT-qPCR) to measure HBV transcripts, two-photon microscopy and iron quantification in different fractions of the 3D liver model.
(DOCX)
