# Supplementary material for: Human Liver Infection in a Dish: Easy-To-Build 3D Liver Models for Studying Microbial Infection
Source: PLoS One. 2016 Feb 10;11(2):e0148667. doi: 10.1371/journal.pone.0148667 (PMC4749187; doi:10.1371/journal.pone.0148667)
Supplement: S1 File — Method details on the HBV production and infection, RT-qPCR) to measure HBV transcripts, two-photon microscopy and iron quantification in different fractions of the 3D liver model. (DOCX) [file pone.0148667.s001.docx]

**S1. Supporting Information**

**HBV production and infection**

The HepAD38 cell line is derived from HepG2 cells and contains the HBV genome (subtype ayw) under tetracycline control [1]. HepG2 H1.3∆X cells are derived from HepG2 cells and contain a stably integrated 1.3-fold HBV genome carrying premature stop codon mutations in both 5’ and 3’ HBx open reading frames [2]. HepG2 H1.3∆X and HepAD38 cells were maintained in DMEM/F-12 with 10% FCS, 3.5 × 10^–7^M hydrocortisone, and 5μg/ml insulin. For HBV particles production, HepAD38 or HepG2 H1.3ΔX cells were grown in Williams E medium with 5% FCS, 7 x 10^-5^M hydrocortisone hemisuccinate, 5µg/ml insulin and 2% DMSO. HBV particles were concentrated from the clarified supernatant by overnight precipitation with 5% polyethylene glycol 8000 (PEG-8000) and centrifugation at 4°C for 60min at 5000rpm. Enveloped DNA-containing viral particles were titered by immunoprecipitation with an anti-PreS1 antibody (kindly provided by C. Sureau, National Institute for Blood Transfusion, Paris), followed by quantitative PCR (qPCR) quantification of viral relaxed circular (RC) DNA with the primers RC 5' (5'-CACTCTATGGAAGGCGGGTA-3') and RC 3' (5'-TGCTCCAGCTCCTACCTTGT-3').

Huh7-NTCP cells were infected as previously described [3] 2D cultures or 3D LSEC-Huh7-NTCP models (setup-4) were infected with normalized amounts of virus at a MOI of 20 genome equivalents/cell in complete DMEM supplemented (except when indicated) with 4% PEG-8000. After overnight incubation the medium was changed and replaced by complete DMEM. The medium of the 3D liver models was then changed daily. Cells were harvested 4days post infection for RNA extraction.

**Quantitative RT-PCR (RT-qPCR) to measure HBV transcripts**

Quantitative PCR (qPCR) was performed using SybrGreen PCR Master mix (Applied Biosystems) and a standard protocol. The primers HBV-trans1s (5'-GCTTTCACTTTCTCGCCAAC-3') and HBV-trans2as (5'-GAGTTCCGCAGTATGGATCG-3') were used to amplify all HBV transcripts except the 0.8 Kb transcript encoding HBx. Rhot2 was used as a reference gene because of its low variation coefficient in human liver tumors and cell lines [4] Rhot2 transcripts were analyzed using the primers Rhot2 s (5'-CTGCGGACTATCTCTCCCCTC-3') and Rhot2 as (5'-AAAAGGCTTTGCAGCTCCAC-3'). Assays were performed in triplicate. Values were calculated according to the ΔCt quantification method with ΔCt = Ct HBV – Ct Rhot2. Results are expressed as the average of at least three independent experiments.

**Two-photon microscopy**

Two-photon microscopy (multiphoton microscope LSM710_NLO upright) was used for visualization and acquisition of 3D images. Hepatic cells were labelled with 2.5μM red cell tracker (CMTPX, Invitrogen C34552; 30min pre-incubation) and the COL-I matrix fibres were detected by the second harmonic generation (SHG) signal (Camelion laser, λ=800nm). To decrease background fluorescence the samples were incubated 12h prior to analysis with complete DMEM medium without phenol red and images acquired in the same medium without serum.

**Quantification of iron in the different fractions of the 3D liver models**

The ferrozine method as described by Hernandez-Cuevas and collaborators [5] was used to quantify iron concentrations at the supernadant or cell lysed fractions from the 3D liver model in the presence or absence of cells. Each sample's iron content was calculated with respect to a standard curve established with 0 to 300 µM FeCl_3_ in the same reaction mixture. Reported values correspond to the mean of experiments performed in triplicate.

**References**

1. Ladner SK, Otto MJ, Barker CS, Zaifert K, Wang G-H, Guo J-T, et al. Inducible expression of human hepatitis B virus (HBV) in stably transfected hepatoblastoma cells: a novel system for screening potential inhibitors of HBV replication. Antimicrob Agents Chemoter. 1997;41: 1715–1720.

2. Lucifora J, Arzberger S, Durantel D, Belloni L, Strubin M, Levrero M, et al. Hepatitis B virus X protein is essential to initiate and maintain virus replication after infection. J Hepatol. European Association for the Study of the Liver; 2011;55: 996–1003.

3. Ni Y, Lempp F a., Mehrle S, Nkongolo S, Kaufman C, Fälth M, et al. Hepatitis B and D viruses exploit sodium taurocholate co-transporting polypeptide for species-specific entry into hepatocytes. Gastroenterology. 2014;146: 1070–1083.

4. Cairo S, Armengol C, De Reyniès A, Wei Y, Thomas E, Renard CA, et al. Hepatic Stem-like Phenotype and Interplay of Wnt/β-Catenin and Myc Signaling in Aggressive Childhood Liver Cancer. Cancer Cell. 2008;14: 471–484.

5. Hernández-Cuevas NA, Weber C, Hon C-C, Guillen N. Gene Expression Profiling in Entamoeba histolytica Identifies Key Components in Iron Uptake and Metabolism. PLoS One. 2014;9: e107102.
